# Plasma Exchange or Immunoadsorption in Demyelinating Diseases: A Meta-Analysis

**DOI:** 10.3390/jcm9051597

**Published:** 2020-05-25

**Authors:** Mark Lipphardt, Manuel Wallbach, Michael J. Koziolek

**Affiliations:** Department of Nephrology and Rheumatology, University Medical Center Göttingen, Robert-Koch-Str. 40, D-37075 Goettingen, Germany; mark.lipphardt@med.uni-goettingen.de (M.L.); manuel.wallbach@med.uni-goettingen.de (M.W.)

**Keywords:** multiple sclerosis, plasma exchange, immunoadsorption

## Abstract

Multiple sclerosis (MS) is an inflammatory disease mainly affecting the central nervous system. In MS, abnormal immune mechanisms induce acute inflammation, demyelination, axonal loss, and the formation of central nervous system plaques. The long-term treatment involves options to modify the disease progression, whereas the treatment for the acute relapse has its focus in the administration of high-dose intravenous methylprednisolone (up to 1000 mg daily) over a period of three to five days as a first step. If symptoms of the acute relapse persist, it is defined as glucocorticosteroid-unresponsive, and immunomodulation by apheresis is recommended. However, several national and international guidelines have no uniform recommendations on using plasma exchange (PE) nor immunoadsorption (IA) in this case. A systematic review and meta-analysis was conducted, including observational studies or randomized controlled trials that investigated the effect of PE or IA on different courses of MS and neuromyelitis optica (NMO). One thousand, three hundred and eighty-three patients were included in the evaluation. Therapy response in relapsing-remitting MS and clinically isolated syndrome was 76.6% (95%CI 63.7–89.8%) in PE- and 80.6% (95%CI 69.3–91.8%) in IA-treated patients. Based on the recent literature, PE and IA may be considered as equal treatment possibilities in patients suffering from acute, glucocorticosteroid-unresponsive MS relapses.

## 1. Introduction

Multiple sclerosis (MS) is a disease which is defined as an inflammatory condition affecting the central nervous system. Its main course of damage is due to abnormal immune mechanisms, resulting in acute inflammation, demyelination, axonal loss, and the formation of central nervous systemplaques consisting of inflammatory cells [1,2].

The epidemiology of MS differs greatly depending on the geographic regions with a prevalence from high levels in North America and Europe (>100/100,000 inhabitants) to low rates in Eastern Asia and sub-Saharan Africa (2/100,000 population). Women are generally more affected than men [3].

Symptoms that occur with the onset of MS are very unspecific, since MS can affect all regions of the central nervous system and can make it hard for a physician to make an early diagnosis. Symptoms of MS include vision problems with a decreased visual acuity (VA) and a prolonged visual evoked potential (VEP), weakness, fatigue, spasms, ataxia, cognitive dysfunction, or numbness [4]. The occurrence of an optic neuritis in its typical form is considered to be associated with MS. However, it is also regarded as a demyelinating clinically isolated syndrome (CIS) with the risk to convert to MS, especially in the white population [5]. With such a variety of symptoms a thorough medical history and examination is essential to make the right diagnosis of MS. Blood tests, lumbar punctures, magnetic resonance imaging, and evoked potential tests help in the process of differentiating between other diseases [6]. Based on the symptoms and the progression of the disease MS is divided in four types: Relapsing-Remitting MS (RRMS), Secondary-Progressive MS (SPMS), Primary-Progressive MS (PPMS), and Progressive-Relapsing MS (PRMS).

MS can be characterized as a T-cell-driven disease with T helper (Th) cells, especially Th-1, Th-2, and Th-17 cells, as the main players in a various inflammatory cascade [7]. For instance, Th-1 cells are responsible for producing Interferon gamma (IFNγ) and tumor necrosis factor alpha (TNF-α) [8]. With the secretion of IFNy and TNF-α inflammation can be maintained by inhibiting Th-2 cell differentiation, since Th-2 cells produce anti-inflammatory cytokines like interleukin (IL)-4 and IL-13 [9,10]. Th-17 cells stimulate inflammation via secreting a vast number of various cytokines like IL-17, IL-21, IL-22, and IL-26 [11,12,13]. As a counterpart regulatory T (Treg) cells inhibit autoimmune responses [14]. In addition to that immunoglobulins (Ig) (especially IgG) are important in the pathogenesis of MS. Evidence of intrathecal Ig production and oligoclonal IgG bands contribute to the diagnosis of MS. Further differentiation shows various types of specific autoantibodies against myelin in subgroups of patients with MS, e.g., anti-myelin oligo-dendrocyte glycoprotein (anti-MOG) or anti-myelin basic protein (anti-MBP) [15]. Antibody-producing B-cells traveling between CNS, blood, and peripheral lymphatic organs clonally expanded B-cells and aggregated B-cells in meninges corroborate a pathophysiological role of B-cells and/or humoral immune answer in the pathogenesis of MS [16,17,18,19].

Based on the myelin protein loss, the geography and extension of plaques, the patterns of oligodendrocyte destruction, and the immunohistopathological evidence of complement activation Lucchinetti et al. described four different immunohistopathological patterns of demyelination in MS [20]. Patterns I and II showed close similarities to T-cell-mediated or T-cell plus antibody-mediated autoimmune encephalomyelitis. Patterns III and IV on the other hand were highly suggestive of a primary oligodendrocyte dystrophy.

Neuromyelitis optica (NMO) on the other hand is described as an idiopathic, severe, demyelinating disease of the central nervous system with the preference to affect the optic nerve and spinal cord. NMO has been considered as a variant of MS. However, with the analysis of clinical, laboratory, immunological, and pathological data the difference to MS is now acknowledged [21].

The treatment regime can be divided in treatment to modify the disease progression and treatment for the acute relapse. In the latter, the administration of high-dose intravenous methylprednisolone (up to 1000 mg daily) over a period of three to five days usually represents the first step in acute MS relapse treatment. A higher second high-dose intravenous methylprednisolone pulse with up to 2 g can be considered in unresponsive patients after an interval of 2 weeks [22,23,24]. Glucocorticoids may downregulate cellular cytotoxicity and lead to the death of activated B cells, but they will not modulate tissue destruction or conduction blockade by local antibody deposition [25]. If symptoms persist, the relapse is defined as glucocorticosteroid-unresponsive and immunomodulation by apheresis is recommended. However, several national and international guidelines have no uniform recommendations on using plasma exchange (PE) or immunoadsorption (IA) in this case. The American Society for Apheresis (ASFA) recommends PE for treatment to category II (“apheresis accepted as second-line therapy”) and IA for treatment to category III (“optimum role of apheresis therapy is not established”) [26]. The American Academy of Neurology also advises the use of PE for adjunctive treatment of relapsing forms of MS (Level B), while IA is not addressed [27,28]. The German guidelines are currently under reconstruction but formerly recommended both procedures as equivalent [29].

In this current issue, we review the use of IA and PE in treating, especially, the acute relapse of MS.

## 2. Effects of Apheresis Therapy

During PE, the patient’s plasma, including all plasma proteins, is removed and substituted by human albumin solution or fresh frozen plasma. The concept of IA involves a selective elimination of plasma proteins, e.g., antibodies, while sparing other plasma proteins [30]. Both techniques include an extracorporeal circulation circuit with systemic and/or local anticoagulation, as well as the need of a vascular access. The latter can either be peripheral venous, if individual vascular situation allows it, or by a central venous catheter. In IA, a secondary circuit is established in which a defined physico-chemical interaction of selected plasma proteins with a defined matrix should theoretically guarantee selective removal of circulating pathogens. In praxis, a bandwidth of proteins are removed [31,32] which are responsible for therapeutic effects but also possible side effects of IA. These effects differ with regard to used matrix of the adsorber, which physicians should be aware.

The exact mechanism by which apheresis treatment works is actually not fully understood. MS patients may benefit by the immediate removal of plasma antibodies, immune complexes and cytokines, induction of a redistribution of antibodies from the extravascular space, and subsequent immunomodulatory changes [30]. Here, cell types with receptors for immunoglobulins (Fc receptors), such as monocytes, macrophages, and natural killer cells, are especially of interest [25]. Besides effects on humoral immune system, experimental data suggest a reduction of circulating autoantigens and regulatory proteins [32] and induction of a higher relative quantity of Treg to Th17 cells [33], as well as a silencing of cellular autoimmune response [32].

Early active MS lesions with an immunohistopathological type II pattern, which are selectively associated with Ig’s and complement deposited along myelin sheaths, predict the best response to apheresis therapy in patients with steroid-unresponsive relapse [34], corroborating the hypothesis of effects on humoral immune response.

## 3. Plasma Exchange

### 3.1. Multiple Sclerosis (with Relapsing-Remitting and Progressive MS Sub-Sections)

The first study comparing the normal therapy regime with PE was performed by Khatri et al. in 1985 and included fifty-four patients with chronic progressive MS [35]. The results showed that patients with the additional PE have a higher improvement rate than patients with a “sham” PE. Following the study of Khatri et al., Weiner et al. enrolled 116 patients in a multicenter, randomized, double-blinded, controlled trial of 11 PE treatments in acute exacerbations of MS [36]. One of the main results showed patients treated with PE to have a significantly enhanced improvement after four weeks. In 1999, a study group of the Mayo Clinic conducted a randomized, sham-controlled, double-blinded study of PE in MS patients with severe neurological deficits after acute relapses, unresponsive to corticosteroids [37]. This study resulted in a moderate to greater improvement in neurological deficits in 42.1% of patients with true PE versus 5.9% of patients with sham PE. With the improved work with PE in the clinical setting, a variety of retrospective studies could demonstrate an improvement rate between 59–87.5% [38,39,40]. In a large study with 153 patients enrolled, Magana et al. identified 90 patients with moderate to marked functional neurological improvement within 6 months after treatment with PE [41].

An excellent and actual overview on apheresis in progressive MS forms is available in Reference [30]. So far, the ASFA recommends PE for treatment to category III: “Optimum role of apheresis therapy is not established. Decision making should be individualized” [26].

### 3.2. Clinically Isolated Syndrome

More recent studies set their focus not only on the relapsing-remitting and progressive MS sub-sections but also on the clinically isolated syndrome [42,43,44]. Therapy response rates ranged between 72–76%, therefore achieving a clinical response in the majority of patients.

### 3.3. Optic Neuritis

Studies analyzing the use of PE in the setting of for severe steroid unresponsive optic neuritis were performed by Ruprecht et al. and Deschamps et al. [45,46]. Ruprecht et al. al. demonstrated an improvement of visual acuity in 70% of patients. Out of these seven patients, three continued to improve with their visual acuity, two remained at a stable state, whereas two patients suffered from worsening symptoms during the follow-ups [46].

In the study performed by Deschamps et al., thirty-four patients with a remaining visual acuity of 0.1 were treated with PE. Afterwards, the median visual acuity was 0.8 [45].

Studies on PE are summarized in Table 1. However, the reader must be aware that the comparability of the studies is limited by the different technical implementation of PE. This varied in frequency, treated plasma volume, and total number of PEs. As a result, the ASFA defined a corridor of technical implementation that recommended treatment of 1–1.5-fold plasma volume per session for a number of 5 to 7 treatments over a period of 10 to 14 days [26].

## 4. Immunoadsorption

### 4.1. Multiple Sclerosis (with Relapsing-Remitting and Progressive MS Sub-Sections)

IA was firstly introduced in the treatment of MS by de Andres et al. in 2000 [69]. They managed a prompt and unequivocal clinical response with a parallel decrease in IgG, fibrinogen, and C3 complement plasma levels in all three patients treated with IA. In the following years, retrospective studies confirmed the initial results of de Andres et al., showing improvement rates from 85–88.3% in MS patients receiving an IA therapy [70,71].

### 4.2. Clinically Isolated Syndrome

Studies incorporating patients with clinically isolated syndrome showed marked to moderate clinical response with a total gain of function in 66–100% of patients after treatment with immunoadsorption [42,72].

### 4.3. Neuromyelitis Optica

The first prospective study investigating effects of IA therapy in patients with MS with steroid-refractory optical neuritis showed an improvement of the mean visual acuity in 8 from 11 patients at day 180 ± 10 after IA [32]. A more recent study confirmed the efficacy and good tolerance of IA in relapses of MS patients with failure to respond to a steroid pulse therapy adequately. Moreover, the study established IA as first-line relapse treatment during pregnancy and breastfeeding [73].

The most commonly used column was a tryptophane-linked polyvinyl alcohol adsorber, but also a Sepharose-conjugated sheep antibodies to human IgG, as well as protein A column, have been used. Table 2 gives an overview about IA-studies in acute relapses of MS.

## 5. Plasma Exchange vs. Immunoadsorption

### 5.1. Multiple Sclerosis (with Relapsing-Remitting and Progressive MS Sub-Sections)

Recently, studies have been designed to compare the efficacy of PE versus IA. The most impressive work is that of Dorst et al. [42]. Sixty-one patients with acute relapse of multiple sclerosis or clinically isolated syndrome and without complete clinical remission of symptoms after at least one cycle of high-dose intravenous methylprednisolone were randomly assigned to receive IA (*n* = 31) or PE (*n* = 30). In the IA group (using a protein A adsorber), the 2.0-fold individual total plasma volume was processed on day 1, and the 2.5-fold on days 2–5. In the PE group, 2 L of plasma (corresponding to the 0.69 ± 0.12-fold individual total plasma volume) were removed each day and substituted by 5% human albumin solution. The median improvement of Multiple Sclerosis Functional Composite after 4 weeks compared to baseline was 0.385 (interquartile range (IQR) 0.200–0.675; *p* < 0.001) in the IA group and 0.265 (IQR 0.100–0.408; *p* < 0.001) in the PE group. Improvement in the IA group was significantly larger (*p* = 0.034) compared to PE. Response rates after 4 weeks were 86.7% in the IA group and 76.7% in the PE group. One deep venous thrombosis occurred in each group. One limitation in interpretation of this study, however, is that the apheresis dose applied was quite different in the two treatment arms and the observation period was relatively short.

Hohenstein et al. reported the successful use of IA with regenerating adsorbers in MS patients as a single center experience [78]. Faissner et al. compared PE and IA directly and demonstrated in a grouped analysis of patients treated with combined PE/ IA, PE, or IA alone, that all groups presented with a better result of visual evoked potentials, providing a valid treatment option in steroid-refractory MS-relapses [50].

### 5.2. Clinically Isolated Syndrome

Dorst et al. [42] also enrolled patients suffering from a clinically isolated syndrome in their recent study. The results are discussed above.

### 5.3. Neuromyelitis Optica

In a small cohort study, Faissner et al. showed equivalent results treating patients with neuromyelitis optica spectrum disorder with IA instead of PE, constituting IA as a valid therapeutic option [77]. Studies of our own also indicate PE and IA to be of equal efficacy and treatment safety [44,79]. We assessed 140 adult patients treated with PE (*n* = 73) or IA (*n* = 67) in steroid refractory multiple sclerosis or neuromyelitis optica. During our studies, we became aware of the fact that differences in body-mass-index, duration of disease, number of treatments, vascular access and treated plasma volumes between IA - and PE cohorts are a main concern for possible bias in the assessment of IA and PE as a treatment for MS patients. We also performed a retrospective single-center cohort study of pediatric patients with inflammatory CNS demyelinating disorders showing excellent tolerance and favorable outcomes of PE and IA in all pediatric patients [31].

## 6. Meta-Analysis on Apheresis Effects on Demyelinating Diseases

### 6.1. Search Strategy and Inclusion Criteria

**Figure 1:**
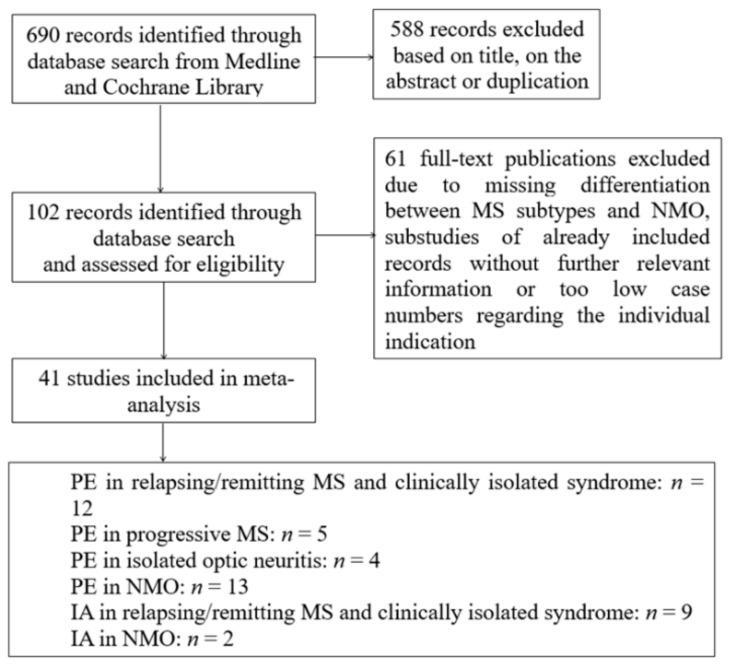
Flow chart of study selection. MS = multiple sclerosis, NMO = neuromyelitis optica, PE = plasma exchange, IA = immunoadsorption.

A systematic search was performed using Medline and Cochrane Library with combinations of the search terms “plasma exchange” OR “immunoadsorption” in combination with the terms “multiple sclerosis” OR “clinical isolated syndrome” OR “neuromyelitis optica” between 1980 and January 2020. Reports were screened independently for relevance based on title and abstract content by two authors (M.L. and M.J.K.). Randomized-controlled trials, as well as prospective cohort studies and retrospective studies and case series, were included if sufficient information on therapy response of PE or IA was provided. Studies with heterogeneous mixing MS, CIS, and/or NMO patients regarding therapy response were excluded if the treatment response was not specified separately in the individual indications. Moreover, case series with a case number less than five in the individual indication were also excluded. It should be mentioned as a limitation that there was no uniform definition of the term “therapy response” in the selected works and, with the exception of a few studies, the majority was retrospective data collection. The flow chart in Figure 1 summarizes the selection of studies in the meta-analysis.

### 6.2. Statistical Analysis

Analysis was performed using RevMan V.5.3 (Nordic Cochrane Centre, Copenhagen, Denmark, the Cochrane Collaboration, 2014). Data were quantitatively synthesized by an inverse-variance-weighted meta-analysis using a random-effect model because of the presence of heterogeneity. The normal approximation interval (sqrt(*p*(1-*p*)/*n*)) was used to generate the confidence interval for the therapy response rate. For studies where the normal approximation interval was zero, the confidence interval was set to one to calculate the random effect model. The 95% normal approximation confidence interval is provided in the meta-analyses.

## 7. Results

With the present search strategy and assessment of full-texts 690 studies, 40 observational and 1 randomized with a total of 1.383 patients could be analyzed. Figure 1 shows the flow chart of study selection.

**Figure 2:**
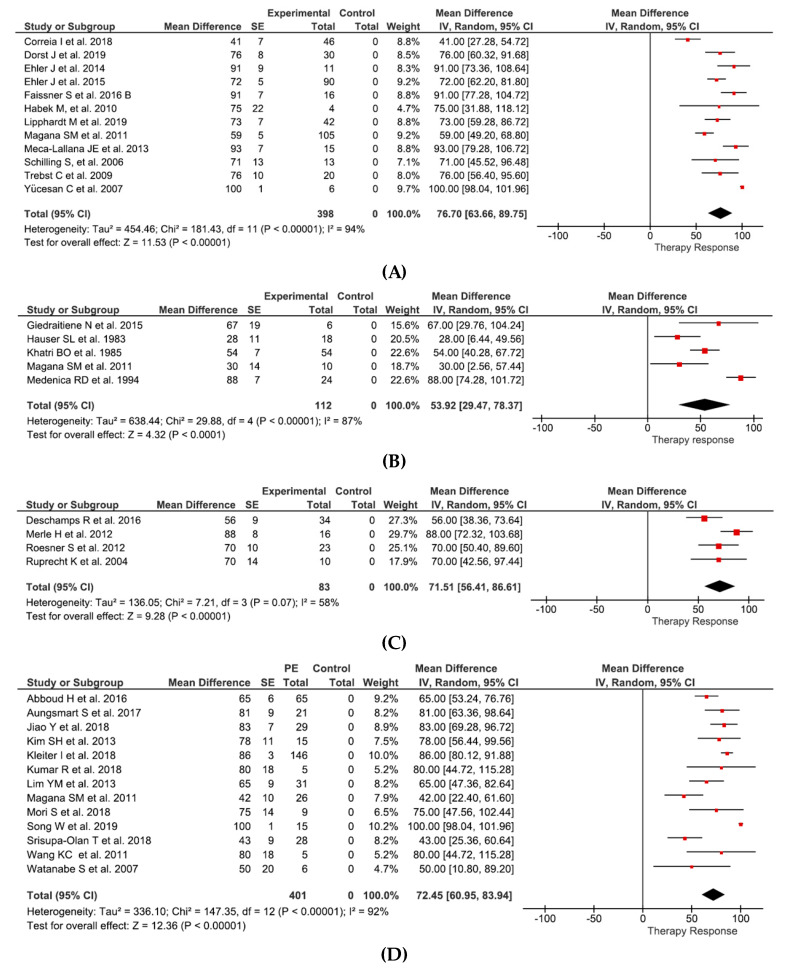

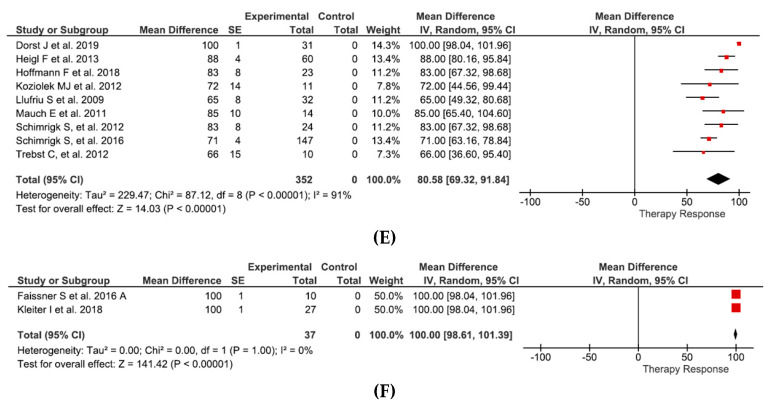
The 95% normal approximation confidence interval is provided in the meta-analyses. The given SE correspond to normal approximation confidence interval (sqrt(*p*(1-*p*)/*n*)). (**A**) Effects of PE in RRMS and CIS. (**B**) Effects of PE in PMS. (**C**) Effects of PE in opticus neuritis. (**D**) Effects of PE in NMO. (**E**) Effects of IA in RRMS and CIS. (**F**) Effects of IA in NMO. RRMS = relapsing-remitting multiple sclerosis, CIS = clinically isolated syndrome, PMS = progressive multiple sclerosis, SE = standard error, IV = instrumental variables. Figure 2A: Correia et al. [51], Dorst et al. [42], Ehler et al. [49], Ehler at al. [43], Faissner et al. [50], Habek et al. [38], Lipphardt et al. [44], Magana et al. [41], Meca-Lallana et al. [48], Schilling et al. [39], Trebst et al. [40], Yücesan et al. [47]. Figure 2B: Giedraitiene et al. [54], Hauser et al. [52], Khatri et al. [35], Magana et al. [41], Medenica et al. [53]. Figure 2C: Deschamps et al. [45], Merle et al. [56], Roesner et al. [55], Ruprecht et al. [46]. Figure 2D: Abboud et al. [61], Aungsmart et al. [62], Jiao et al. [64], Kim et al. [60], Kleiter et al. [67], Kumar et al. [66], Lim et al. [59], Magana et al. [41], Mori et al. [65], Song et al. [68], Srisupa-Olan et al. [63], Wang et al. [58], Watanabe et al. [57]. Figure 2E: Dorst et al. [42], Heigl et al. [70], Hoffmann et al. [73], Koziolek et al. [32], Llufriu et al. [80], Mauch et al. [71], Schimrigk et al. [75], Schimrigk et al. [76], Trebst et al. [72]. Figure 2F: Faissner et al. [77], Kleiter et al. [67].

Effects of PE can be summarized as follows: in relapsing-remitting MS and clinically isolated syndrome (12 studies and 398 patients) therapy response of 76.6% (95%CI 63.7–89.8%) (Figure 2A), in progressive MS (5 studies and 112 patients) therapy response of 53.9% (95%CI 29.5–78.4) (Figure 2B), in isolated optic neuritis (4 studies and 83 patients) therapy response of 71.5% (95%CI 56.4–86.6%) (Figure 2C), and in NMO (13 studies and 401 patients) therapy response of 72.5% (95%CI 61.0–83.9%) (Figure 2D).

Effects of IA can be summarized as follows: in relapsing-remitting MS and clinically isolated syndrome (9 studies and 352 patients), therapy response of 80.6% (95%CI 69.3–91.8%) (Figure 2E); and in NMO (2 studies and 37 patients), therapy response of 100% (95%CI 98.6–101.4%) (Figure 2F).

## 8. Safety Profile

### 8.1. General

Another important fact to consider is the treatment safety. The noted rates of side effects during those apheresis treatments are very heterogeneous. In the literature one can find complication rates from 4.2% until 25.6% [81,82,83,84]. In 2011, Köhler et al. postulated lower side effects using IA in patients suffering from myasthenia gravis [85]. They claim that a possible reason for the difference was due to the absence of albumin-substitution. Zoellner et al. designed a study to investigate the fibrinogen level and the occurrence of bleeding complications [86]. They demonstrated IA to have a lower degree of fibrinogen reduction as PE. Bleeding complications occurred in 1.3–3.1% of treatments. Schneider-Gold et al. reported allergic reactions, hypocoagulability, and bronchorespiratory infections with a significant higher frequency in the PE-only group as compared to the IA-only group or the both combined [87].

### 8.2. Multiple Sclerosis (with Relapsing-Remitting and Progressive MS Sub-Sections) and Clinically Isolated Syndrome

In the recent study performed by Dorst et al. [42], a general well tolerance was observed with 5 mild infections in the PE group and 4 mild allergic reactions in the IA group. Furthermore, courses of anemia and thrombocytopenia were documented with anemia being more frequent in the PE group and thrombocytopenia being more frequent in the IA group.

### 8.3. Multiple Sclerosis (with Relapsing-Remitting and Progressive MS Sub-Sections) and Neuromyelitis Optica

In our studies the complication rate was about 3.7% in over 780 apheresis cycles. Furthermore, we could not detect any differences regarding the safety profile of IA versus PE [44,79].

All in all, both IA and PE have a high tolerability regarding the safety profile. It should be added that the majority of the documented side effects are to be considered as mild. However, the use of IA and PE should be reserved to specialized centers familiar with technical procedure and experienced with this specialized patient population to ensure a high quality of treatment with low complication rates.

## 9. Treatment Predictors

### 9.1. General

One major predicting factor is the time to initiate apheresis treatment. Early initiation of apheresis correlates with a higher response rate as was shown by several study groups [44,60,80,88]. In the onset of sudden hearing loss, the early initiation of apheresis treatment was also beneficial [89].

Comparing the cumulative corticosteroid doses in apheresis-responders versus non-responders, no significant difference was shown, which makes a synergistic effect of apheresis and corticosteroids unlikely [44].

### 9.2. Multiple Sclerosis (with Relapsing-Remitting and Progressive MS Sub-Sections)

Magana et al. postulated the duration of the disease and preserved deep tendon reflexes as important clinical predictors [41]. A different approach was followed by the study group of Stork et al., who conducted a single-center cohort study with 69 MS patients, evaluating treatment response in relation to histopathologically defined immunopathological patterns of MS [34]. As early active demyelinating MS lesions can be divided in 3 different immunopathological patterns of demyelination, Stork et al. demonstrated that patients with pattern 1 and 2 are most likely to benefit from apheresis treatment, especially in patients with pattern 2 who show signs of a humoral immune response in particular. Patients with pattern 3 most likely do not benefit from apheresis treatment. During our studies, we also became aware of the fact that patients having a good response to apheresis treatment were significantly younger than non-responders [44]. This observation may be due to a decrease in remyelination efficiency, as proposed by Sim et al. [90]. A gender-related treatment benefit towards the female gender was identified in sub-groups of MS patients [44,91].

### 9.3. Neuromyelitis Optica

In a large study performed by Kleiter et al., it was shown that PE or IA exerts a better recovery from acute relapses in patients suffering from neuromyelitis optica if they had isolated myelitis [92]. More recent studies focused on the plasma anti-aquaporin-4 immunoglobulin G antibody as a positive predictor for treatment success with PE or IA in patients suffering from neuromyelitis optica spectrum disorder [12]. In both studies, particularly, patients with a positive anti-aquaporin-4 immunoglobulin G antibody responded well to the treatment with PE and IA. In addition to that, no advantage was revealed for either PE or IA. The disease specificity of anti-aquaporin-4 immunoglobulin G antibody is almost at 100% and clinical studies with immunohistochemical evidence suggest that this antibody plays a central role in the pathogenesis of neuromyelitis optica spectrum disorder [93].

These predictors can thus be summarized according to various variables. Table 3 provides a compilation.

## 10. Therapeutic Efficacy and Time Course

As for the time course of the therapeutic effect, the current literature agrees on regular neurological follow-ups after 6 months, manifesting a continuous and maximal clinical effect of the apheresis treatment [41,44,60,80]. Therapeutic effects over such a long period of time suggest immunomodulatory actions of apheresis rather than antibody removal on its own [95]. Those immunomodulatory actions happen most likely at the level of Th-cells and CNS-associated proteins, like the myelin basic protein. The prolonged therapeutic effect can be thought of as a clinical correlate of the immunomodulatory components of therapeutic apheresis. Furthermore, the duration of the apheresis induced therapeutic effect can be involved in the treatment process of initiating or changing disease-modifying drugs.

## 11. Conclusions

The focus of this current issue is the use and comparison of immunoadsorption and plasma exchange in the treatment of multiple sclerosis with the main concern of acute relapses.

Based on the studies of the current literature and performance of a meta-analysis, including 690 studies, 40 observational and 1 randomized with a total of 1383 patients, plasma exchange and immunoadsorption are treatment options of equal effectivity for acute glucocorticosteroid-unresponsive multiple sclerosis relapses.

For the meta-analysis randomized-controlled trials, prospective cohort studies, retrospective studies, and case series with sufficient information on therapy response of plasma exchange or immunoadsorption were included. Studies with heterogeneous mixing multiple sclerosis, clinically isolated syndrome, and/or neuromyelitis optica patients regarding therapy response were not included if the treatment response was not specified separately in the individual indications. 

Plasma exchange has a therapy response of 76.6% in relapsing-remitting multiple sclerosis (RRMS) and clinically isolated syndrome (CIS), 53.9% in progressive multiple sclerosis (PMS), 71.5% in isolated optic neuritis, and 72.5% in neuromyelitis optica (NMO). Immunoadsorption (IA) has a therapy response of 80.6% in relapsing-remitting multiple sclerosis and clinically isolated syndrome and 100% in neuromyelitis optica. 

Early treatment initiation with a median of 2–3 weeks and a patient age below 50 are considered to be beneficial regarding a treatment success. In addition to that, a treatment count of 5 to 7 with one plasma volume is also beneficial for treatment success, whereas patients suffering from progressive multiple sclerosis have a lower beneficial rate of apheresis therapy. Both immunoadsorption and plasma exchange have a high safety profile and a high tolerability regarding side effects.

Nevertheless, data situation is too heterogeneous regarding procedures and technical implementation to be finally assessed.

## Figures and Tables

**Table 1:** Studies on plasma exchange (PE) in treatment of relapsing-remitting multiple sclerosis (RRMS), clinically isolated syndrome (CIS), progressive MS, isolated optic neuritis, and neuromyelitis optica (NMO). EDSS = Expanded Disability Status Scale.

**“Relapsing-Remitting Multiple Sclerosis” and “Clinically Isolated Syndrome”**							
**Citation**	**Year**	***n***	**Design**	**No. of Treatments**	**Treated Plasma Volume (mL)**	**Outcome**	**Limitation**
[36]	1989	116	Double-blind, multi-center, randomized	11	n.a.	Significant improvement after 4 weeks	No plasmapheresis protocol specifications
[37]	1999	36	Double-blind	7	3000	Therapy response in 42% of patients	Patient collective with heterogenous MS-types
[39]	2005	13	Retrospective	5	3000	Therapy response in 71% of patients	Small number of subjects
[47]	2007	6	Retrospective	4	1.0-fold plasma volume	Therapy response in 100% of patients	Small number of subjects
[40]	2009	20	Retrospective	3–7	1.5-fold plasma volume	Therapy response in 76% of patients regarding visual acuity	Small number of subjects
[38]	2010	4	Retrospective	5	2750	Therapy response in 75% of patients	no placebo, Small number of subjects, the study was observational in character
[41]	2011	153	Retrospective	7	n.a.	Therapy response in 59% of patients	Patient collective with heterogenous MS-types
[48]	2013	15	Retrospective	≥7	1.0-fold plasma volume	Therapy response in 93.3% of patients	RRMS + CIS
[49]	2014	11	Retrospective	Median 7 (3–8)	3000 (2200–3500)	Therapy response in 91% of patients	CIS only
[43]	2015	90	Retrospective	3–8	1.0-fold plasma volume	Therapy response in 72% of patients	The lack of a control group
[50]	2016	16	Retrospective	n.a.	2000	Therapy response in 91% of patients regarding visual evoked potential	Small number of subjects and a higher expanded disability status scale in patients in the PE only group
[51]	2018	46	Retrospective	Mean 7.39 sessions	n.a.	Complete therapy response in 41% of patients and partial therapy response in 39% of patients	Patient collective with heterogenous MS-types
[44]	2019	42	Retrospective	4–11	Mean 2930 median 2000	Therapy response in 73% of patients	patients without sufficient follow-up data had a significantly higher patient age and longer duration of disease
[42]	2019	30	Double-blind, randomized, uni-center	On 5 days	0.69 ± 0.12-fold individual total plasma volume	Therapy response in 76% of patients	Lack of blinding and small number of subjects
**“Progressive Multiple Sclerosis”**							
**Citation**	**Year**	**n**	**Design**	**No. of Treatments**	**Treated Plasma Volume (mL)**	**Outcome**	**Limitation**
[52]	1983	18	Prospective, randomized	4–5	n.a.	Therapy response in 27.8% of patients	Small number of subjects, no plasmapheresis protocol specifications
[35]	1985	54	Double-blind controlled	20	n.a.	Therapy response in 54% of patients	No plasmapheresis protocol specifications
[53]	1994	24	Prospective	8	n.a.	Therapy response in 87.5% of patients	Small number of subjects, no plasmapheresis protocol specifications
[41]	2011	10	Retrospective	7	n.a.	Therapy response in 30% of patients	Small number of subjects
[54]	2015	6	open-label, single-center proof of concept study	4	2000–2500	Therapy response in 66.7% of patients	Small number of subjects
**“Isolated Optic Neuritis”**							
**Citation**	**Year**	***n***	**Design**	**No. of Treatments**	**Treated Plasma Volume (mL)**	**Outcome**	**Limitation**
[46]	2004	10	Retrospective	n.a.	n.a.	Therapy response in 70% of patients	Small number of subjects
[55]	2012	23	Retrospective	5	~3000	Therapy response in 70% of patients	heterogenous
[56]	2012	16	Retrospective	5	1.0-fold plasma volume	Therapy response in 87.5% of patients	Small number of subjects
[45]	2016	34	Retrospective	Median 5, range 5–10	1.5-fold body mass volume	Therapy response in 56% of patients regarding visual acuity	The lack of a control group
**“Neuromyelitis Optica”**							
**Citation**	**Year**	***n***	**Design**	**No. of Treatments**	**Treated Plasma Volume (mL)**	**Outcome**	**Limitation**
[57]	2007	6	Retrospective	3–5	2000–3000	Therapy response in 50% of patients	Small number of subjects
[58]	2011	5	Retrospective	≥5	1.0-fold plasma volume	Therapy response in 80% of patients	Small number of subjects
[41]	2011	26	Retrospective	7	n.a.	Therapy response in 42.3% of patients	Historical cohort study
[59]	2013	31	Retrospective	n.a.	n.a.	Therapy response in 65% of patients	No study controlled treatment regimes
[60]	2013	15	Retrospective	6	1.0–1.5-fold plasma volume	Therapy response in 78% of patients	Small number of subjects
[61]	2016	65	Retrospective	5–7	1.5-fold plasma volume	Therapy response in 65% of patients	Selection bias; use of EDSS scores as the primary outcome measure
[62]	2017	21	Retrospective	5	n.a.	Therapy response in 81% of patients	Use of EDSS scores as the primary outcome measure
[63]	2018	28	Retrospective	5	1000	Therapy response in 42.9% of patients	Use of EDSS scores as the primary outcome measure
[64]	2018	29	Retrospective	2–7	1.0-fold plasma volume	Therapy response in 82.8% of patients	Heterogenous treatment protocols
[65]	2018	9	Retrospective	7	1.0-fold plasma volume	Therapy response in 75% of patients	Small number of subjects
[66]	2018	5	Retrospective	5 (3–7)	1.0-fold plasma volume	Therapy response in 80% of patients	Small number of subjects
[67]	2018	146	Retrospective	≥3	n.a.	Therapy response in 86% of patients	Heterogenous treatment protocols
[68]	2019	15	Retrospective	2–3	n.a	Therapy response in 100% of patients	Small number of subjects

**Table 2:** Studies on immunoadsorption in treatment of relapsing-remitting multiple sclerosis (RRMS), clinically isolated syndrome (CIS) and neuromyelitis optica (NMO).

**“RRMS” and “CIS”**								
**Citation**	**Year**	***n***	**Design**	**No. of Treatments**	**Treated Plasma Volume (mL)**	**Matrix of Adsorber**	**Outcome**	**Limitation**
[69]	2000	3	Retrospective	5–6	n.a.	n.a.	Therapy response in 100% of patients	small number of subjects
[74]	2005	12	Prospective	14	1.5-fold plasma volume	Sepharose-conjugated sheep antibodies to human immunoglobulin (IgG)	No significant therapy response	small number of subjects and patient collective with heterogenous MS-types
[71]	2011	14	Retrospective	5–6	n.a.	Tryptophan	Therapy response in 85% of patients	small number of subjects
[75]	2012	24	Retrospective	Mean 5 (range 3–6)	2000–2500	Tryptophan	Therapy response in 83% of patients	small number of subjects and patient collective with heterogenous MS-types
[72]	2012	10	Retrospective	5–7	2500	Tryptophan	Therapy response in 66% of patients	small number of subjects
[32]	2012	11	Prospective	5	2500	Tryptophan	Therapy response in 72% of patients	small number of subjects
[70]	2013	60	Retrospective	6	2000	Tryptophan	Therapy response in 88% of patients	only qualitative data regarding the therapeutic success and clinical data on tolerability were available
[76]	2016	147	Retrospective	n.a.	2000–2500	Tryptophan	Therapy response in 71% of patients	Expanded Disability Status Scale was used to measure a change in relapse-related disability
[73]	2018	23	Retrospective	Mean 5.8	2031 ± 230	Tryptophan	Therapy response in 83% of patients	Lack of a control group; use of immunoadsorption was limited in some study centers
[44]	2019	32	Retrospective	5–7	2000–2500	Tryptophan	Therapy response in 65% of patients	patients without sufficient follow-up data had a significantly higher patient age and longer duration of disease
[42]	2019	31	Prospective, double-blind, randomized, uni-center	On 5 days	2.0-fold total plasma volume on day 1, and the 2.5-fold total plasma volume on day 2–5	protein A	Therapy response in 100% of patients	Lack of blinding and small number of subjects
**“NMO”**								
**Citation**	**Year**	***n***	**Design**	**No. of Treatments**	**Treated Plasma Volume (mL)**	**Matrix of Adsorber**	**Outcome**	**Limitation**
[77]	2016	10	Retrospective	Mean 5.2 (3–7)	2000–2500	Tryptophan	Therapy response in 100% of patients	Small number of subjects
[67]	2018	27	Retrospective	≥3	n.a.	Tryptophan or Protein A	Therapy response in 100% of patients	Heterogenous treatment protocols

**Table 3:** Predictors of apheresis response. EDSS = Expanded Disability Status Scale; MRI = magnetic resonance imaging. * Pediatric patients only.

**“Multiple Sclerosis” (with Relapsing-Remitting and Progressive MS Sub-Sections)**			
**Classification**	**Predictor**	**Citation**	**Meaning**
Clinical signs and symptoms	EDSS ≤ 5	[43]	Indicates good apheresis response
	Preserved deep tendon reflexes	[41]	Indicates good apheresis response
Demographics	Younger age	[44]	Indicates good apheresis response
	Female	[37,91]	Indicates good apheresis response
Histological classification and localization	Gadolinium positive MRI lesions	[43]	Indicates good apheresis response
	Histological type 1 and 2 pattern	[34]	Indicates good apheresis response
	Histological type 3 pattern	[34]	Indicates poor apheresis response
Pre-treatment	No disease modifying drugs	[43]	Indicates good apheresis response
	Short duration of disease	[41]	Indicates good apheresis response
**“Neuromyelitis Optica”**			
**Classification**	**Predictor**	**Citation**	**Meaning**
Histological classification and localization	Isolated myelitis	[85]	Indicates good apheresis response
Laboratory values	Anti-aquaporin-4 IgG positive	[12]	Indicates good apheresis response
**“Mixed”**			
**Classification**	**Predictor**	**Citation**	**Meaning**
Apheresis	Early initiation	[44,60,80,88]	Indicates good apheresis response
Clinical signs and symptoms	Lower baseline scores on the EDSS, visual outcome, and gait scales	[94] *	Indicates good apheresis response
Pre-treatment	Cumulative corticosteroid doses	[44]	Irrelevant for apheresis response

## Abbreviations

ASFA	American society for apheresis
CIS	Clinical isolated syndrome
Ig	Immunoglobulin
IL	Interleukin
IFNγ	Interferon gamma
NMO	Neuromyelitis optica
MS	Multiple Sclerosis
PE	Plasma exchange
PPMS	Primary-Progressive MS
PRMS	Progressive-Relapsing MS
RRMS	Relapsing remitting MS
SPMS	Secondary-Progressive MS
Th	T helper
Treg	T regulatory
TNF-α	Tumor necrosis factor alpha
VA	Visual acuity
VEP	Visual evoked potential
